# Perspectives for immune plasma treatment of COVID-19

**DOI:** 10.3906/sag-2005-410

**Published:** 2021-02-26

**Authors:** Başak SAYINALP, Olgu Erkin ÇINAR, İbrahim Celalettin HAZNEDAROĞLU

**Affiliations:** 1 Department of Internal Medicine, Faculty of Medicine, Hacettepe University, Ankara Turkey; 2 Department of Hematology, Faculty of Medicine, Hacettepe University, Ankara Turkey

**Keywords:** COVID-19, pandemic, convalescent plasma

## Abstract

**Background/aim:**

The SARS-CoV-2 infection was declared as a pandemic by the World Health Organization (WHO) on March 11, 2020, and the death toll from COVID-19, which is the disease caused by SARS-CoV-2, has already surpassed that of many previous epidemics. A wide variety of treatment options are being considered for COVID-19, but there is still no definitive treatment or vaccine. This study aims to explain the background of convalescent plasma (CP) treatment and its relations with COVID-19 immunity, to define ideal treatment procedures, and to reveal present and future perspectives in the light of the rapidly growing data.

**Immunological basis of COVID-19-associated immune response and convalescent plasma as a treatment option:**

Since it has been shown that the impaired immune response of the host is one of the most important factors that increase the severity of the infection, treatment strategies to suppress aberrant immune activation are currently being considered. CP, which is derived from recently recovered patients and contains neutralizing antibodies and many other immunemodulatory substances, seems to be the most convenient strategy to restore normal immune function considering the fast spreading nature of the ongoing pandemic.

**Conclusion:**

Even though mechanisms of action of plasma therapy are not fully delineated, it was shown that it could lead to a reduction in mortality since other alternatives such as monoclonal antibodies or SARS-CoV-2 hyperimmunoglobulin require much more time and effort to be developed.

## 1. Introduction

The term ‘epidemic’ comes from the conjugation of the Greek words ‘epi’ (on) and ‘demos’ (people) and was first mentioned by Homer. Hippocrates first used this term in the field of medicine to describe an assortment of clinical syndromes occurring and spreading at a certain location in a given period [1]. Over time, the term has been utilized to describe outbreaks of single infectious diseases, such as the Bubonic Plague (Black Death), which caused the deaths of approximately 200 million people between 1347 and 13511Visual Capitalist (2020). Visualizing the History of Pandemics [online]. Website u1d8b [accessed 20 June 2020].. On March 11th of 2020, the World Health Organization (WHO) declared that the viral infection and subsequent outbreak ofSARS-CoV-2 constituted a pandemic2World Health Organization (2020). WHO announces COVID-19 outbreak a pandemic [online]. Website u1d90 [accessed 20 June 2020].. ‘Pandemic’ is the term used for epidemicscharacterized by multiple, infectious casesin distinct countries and continents. COVID-19 and the associated immune syndrome is a multisystemic disorder following the development of the SARS-CoV-2 infection. Currently, the death toll of COVID-19 has already surpassed those of previous epidemics such as SARS-CoV-1, MERS, Ebola, Yellow Fever, and the H1N1 influenza virus and mortality numbers are still growing1.

A wide variety of treatment options are currently being considered for the challenging COVID-19-associated immune syndrome. From an immune-biological point of view, the impaired/deficient immune reaction of the host could play a prominent role, particularly in patients presenting with more severe clinical diseases. Drugs and treatment options focusing on immune modulation of the host are currently under development. Convalescent/immune plasma (CP) derived from recently recovered patients is a classical way of passive immunotherapy experienced within the last 80–100 years [2]. Since the SARS-CoV-2 specific hyperimmune immunoglobulin preparation or unique monoclonal antibodies require much more time and effort to be developed, CP, which contains neutralizing antibodies to the relevant pathogen [3], is the most convenient way, considering the fast spreading nature of the ongoing COVID-19 pandemic until an effective vaccine becomes available.

## 2. Immunological basis of COVID-19 associated immune response

Currently, available data indicates that about 80% of SARS-CoV-2 infections are asymptomatic or mildly symptomatic, whereas the remaining 20% can manifest itself as the severe disease form. The host immune system, together with the pathogen virus dose, viral strain, time-dependent viral transmission, and host exposure kinetics determine the severity of the disease. Since it is a newly emerging disease, precise data regarding the immunological aspects of the COVID-19 syndrome are still being collected. The most analogical explanations are being made based on previous members of the coronavirus family viruses, namely SARS-CoV and Milddle East respiratory syndrome (MERS).[4].

Innate immune system and type-1 interferon molecular expressions, along with other proinflammatory cytokines, including IL-1, IL-6, and TNF-alpha, play a central role in the clearance of coronaviruses [5]. The severe disease forms of SARS-CoV, MERS, and SARS-CoV-2 are associated with immune evasion strategies. These biological events enable the viruses to proliferate in the infected host cells. Eventually, the infected cells undergo programmed cellular death. Meanwhile, the viral particles, along with the intracellular components, such as nuclear antigens triggering the innate inflammatory mechanisms, are released into the microenvironment. At this critical biological stage, adaptive immunity with CD4^+^, CD8^+^ T-cells, and antibody-producing B-cells is involved, and a second wave of inflammation takes place [4]. Coronaviruses could also further lead to lymphocyte apoptosis, another well-known strategy for immune evasion [6].

**Figure 1:**
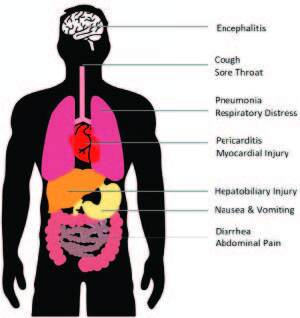
AlthoughCOVID-19 appears primarily as a respiratory disease, many organs/systems are affected due to the direct cytopathic effect of the virus or because of the uncontrolled immune response against it.

In a subpopulation of infected patients with SARS-CoV-2, enhanced immune activation could lead to a cytokine storm, which clearly contributes to disease severity with increased morbidity and even mortality [7]. The reduction in the total number of T-cells was demonstrated in patients with severe COVID-19-associated immune syndrome requiring intensive critical care. T-cell numbers are negatively correlated with the circulating IL-6, IL-10, and TNF-alpha. Patients with a history of recovering fromthe COVID-19 syndromeshowed a reduced concentration in proinflammatory cytokine levels together with increments in the circulating T-lymphocyte counts. Moreover, significant increases in exhaustion markers such as PD-1 were observed on the T-cells, which became more apparent during the clinicobiological course of the COVID-19 associated immune syndrome [8]. Many systems in the body are affected by the direct cytopathic effect of the virus or one/several of these immune mechanisms. Some of the clinical and pathological manifestations are demonstrated in Figure 1. Although there is much to still learn about the immune pathology of this newly emerging syndrome, the impaired and exaggerated immune response seems to be the most important mechanism that increases clinical severity. Therefore, treatment strategies to suppress aberrant immune activation and restore the normal function of the immune system are urgently needed.

## 3. Convalescent/immune plasma therapy as a treatment option

### 3.1. The concept of passive immune transfer

Passive immunotherapy dates back to the late 19th century, when patients with diphtheria were treated with the immune sera of animals such as sheep, goats, or horses. However, the use of human CP has been in practice since the early 20th century with the emergence of viral outbreaks such as the 1918 H1N1 influenza virus pandemic [9]. The immune plasma transfer approach was also used successfully during the 2003 SARS-CoV epidemic [10], the 2009–2010 H1N1 influenza virus pandemic [11], the 2012 MERS epidemic [12], and the 2014 West African Ebola epidemic [13]. In a systematic review and exploratory metaanalysis by Mair-Jenkins et al., the administration ofCP for the treatment of severe acute respiratory infections of viral etiology was assessed. The authors concluded that, in spite of the lack of randomized controlled clinical trials, plasma treatment could lead to a reduction in mortality, especially when it is administered in early periods following the onset of the infection [14]. This observation can be partly explained by the smaller amount of initial inoculum. Since the viral load in the initial inoculum is much lower than that of the established infection, it makes it easier for the passive antibodies to neutralize them. Viral neutralization and relevant cellular immunological mechanisms can operate more efficiently at the beginning of the immune response [15].

### 3.2. Mechanisms of action

Several mechanisms are now being proposed for the mechanism of action ofCP therapy. Neutralizing antibodies, which bind to the virus and block viral entry, fusion, and egress, are the most recognized mechanism [16]. A dose of neutralizing antibodies is correlated with higher viral load reduction and lower mortality, as suggested in previous studies on the Ebola virus and the Junin virus, the causative agent of Argentine hemorrhagic fever [17, 18]. The fact that it contains antibodies that neutralize the virus and block its entrance to host cells by binding to crucial viral antigens is not the only mechanism of action ofCP. Immune plasma therapy may also function via modifying the host inflammatory response and related endogenous substances. 

The efficacy of CP is dependent upon distinct immunobiological actions [19]. Numerous cellular responses are involved in the immunological responses, including T-cells, B-cells, dendritic cells, and macrophages. They are all involved in the ‘cellular immunity effects’ of CP via modulation of cytokines and chemokines within the maturation and activation of each unique cell type. Although these mediators and their clinical effects have not yet been exactly ascribed to direct CP contents and in vivo effects, many postulations have been made regarding the already known mechanisms of IVIg treatment, which acts through similar pathways. Antibody-dependent cellular cytotoxicity, opsonization, and complement activation take part in humoral immunity. These complicated biological processes could aid antibodies in stimulating cellular immunity within the actions of cytokine/chemokine networks [20]. Passive antibody administration failed to protect mice deficient in CD4^+^ T cells, and Th1- and Th2-associated cytokines from infection [21,22]. Likewise, FrCas^E^ retrovirus-infected mice to which the passive antibody was administered had protective immunity in spite of a lower viral inoculum mimicking the neutralizing function of the antibody [23]. Those in vivo observations generated the hypothesis that passive antibody administration could lead to similar immunobiological effects in humans. That hypothesis will be tested while performing future research during the ongoing COVID-19 pandemic because, at present, there is no available and effective vaccine or antiviral drug.

Another important problem in the course of COVID-19 is coagulopathy, which often occurs in findings similar to disseminated intravascular coagulopathy or antiphospholipid antibody syndrome. Critically ill patients exhibited positivity for anticardiolipin IgA antibodies, aswell as for anti-β2-glycoprotein I IgA and IgG antibodies [24].CP therapy may also have a role in terms of blocking responsible antibody for prevention or amelioration of antiphospholipid antibody syndrome-like coagulopathy, just as this has been observed in IVIg therapy.

### 3.3. Clinical indications of the immune plasma therapy

The efficacy of CP therapy has not yet been proven by randomized controlled clinical trials. According to preliminary data, the most beneficial way to use immune plasma therapy is within the first 10 days of the disease course and in COVID-19 patients following severe or life-threatening disease criteria3U.S. Department of Health and Human Services Food and Drug Administration (2021). Investigational COVID-19 Convalescent Plasma [online]. Website u1d92 [accessed 00 Month Year]..

Severe disease criteria:

- Dyspnea

- Respiratory rate ≥30/min

- Arterial oxygen saturation ≤93%

- Pa/FiO_2_ ≤ 300

- >50% progression of lung infiltrates within 24–48 h

Life-threatening disease criteria:

- Respiratory failure

- Sepsis or septic shock

- Multiorgan dysfunction or failure

Informed consent must be obtained from the patient or from the patient’s relatives.

### 3.4. Donor eligibility

Individuals who donate immune plasma must first be in good health; meeting universal blood donation criteria and adhering to standard operating procedures prior to the pandemic are also important. As with many other blood product donations, screening should be done for the main viral infection factors that can be transmitted by blood. The main essentials of those tests include HIV 1-2 and HIV NAT, anti-HCV-HCV NAT, HBs Ag, and VDRL but should also include regional infectious diseases, if any. Special warnings regarding blood donation during the pandemic period have been specifically stated by WHO4World Health Organization (2021). WHO Blood Regulators Network (BRN) Donor Selection in case of Pandemic Situations [online]. Website https://www.fda.gov/media/136798/download [accessed 20 June 2020]..

The following criteria regarding COVID-19 disease must be met5European Commission Directorate-General for Health and Food Safety (2020). An EU Programme of COVID-19 Convalescent Plasma Collection and Transfusion [online]. Website https://www.who.int/bloodproducts/brn/DonorSelectionincaseofPandemicSituations.pdf?ua=1 [accessed 20 June 2020].:

- During the disease period, a diagnostic test must be positive (e.g., SARS-CoV-2 PCR with a nasopharyngeal swab) or a SARS-CoV-2 serological antibody test must be found to be positive after recovery.

- Complete COVID-19 symptoms have to be resolved at least 14 days before donation (there is no need to prove that the PCR test has become negative)

- Donation must be from male donors or nonpregnant female donors or anti-HLA antibody negativity in women with a history of pregnancy

- Testing of anti-SARS-CoV-2 antibody titers, if available:

- Ideally, antibody positivity should be found at a titer of 1:160 or higher according to the FDA and 1:320 or higher according to European Commission on Health and Food Safety. If no alternative is available, a lower titer may also be effective.

- If the test is not available, it is recommended that the plasma sample be stored for testing later when available.

**Figure 2:**
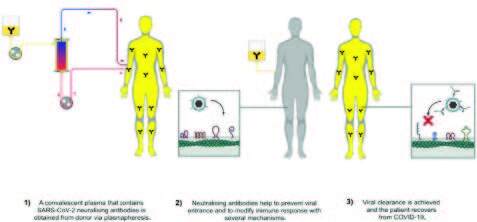
Schematic steps of convalescent plasma treatment.

It is essential that all these processes be carried out in authorized and high standard blood banks. The schematic steps of CP treatment are demonstrated in Figure 2.

### 3.5. Pathogen reduction

Although it is not yet clear whether the SARS-CoV-2 virus is transmitted by blood and since plasma products should not contain any nucleic acid-containing cells (leukocytes or microorganisms), inactivation of possible pathogens (especially for those not covered or detected in screening tests) should be performed in each donated plasma product in centers with the technical abilities and trained personnel. For this, solvent/detergent-based treatments that are very effective against viruses with lipid envelopes or light-based methods targeting the nucleic acid structure (e.g., amotosalen + UV, riboflavin + UV, or methylene blue + visible light) can be used. Pathogen-reduced plasma products have not been observed to cause any toxic or immunologic adverse reactions beyond what would be expected from any other plasma products.

### 3.6. Labeling

It is recommended to label the donor’s ABO and Rh groups, the date of donation, the amount of plasma contained, and the antibody test result for the patient it is prepared for [25].

## 4. Typical clinical application of immune plasma therapy for the management of COVID-19 in a critical case

**Figure 3:**
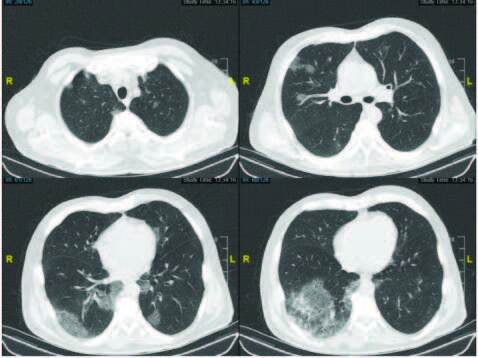
Typical chest computed tomography findings of a patient with COVID-19 pneumonia shows scattered ground glass densities, especially involving subpleural and peripheral regions of the lungs.

A 55-year-old male with a history of myelodysplatic syndrome (MDS) with fluorescent antibody test (FAB) refractory anemia excess blasts-1 subtype (MDS/ RAEB-1), complicated by disseminated systemic tuberculosis and associated kidney disease, was admitted to our hospital with complaints about an ongoing, high fever and persistent cough lasting for about 3 days. He had recently been discharged from the hospital after a follow-up of 2 months for the disseminated tuberculosis infection and was still on the classic four-drug regimen (isoniazid, rifampin, pyrazinamide, and ethambutol). Low-dose chest computed tomography indicated COVID-19 pneumonia (Figure 3.), and the nasopharyngeal swab sample was also found to be positive for the COVID-19 infection; he was then urgently hospitalized. Taking into consideration the immunocompromised status of the multimorbid patient and his worsening status, despite antiviral (favipiravir) treatment and supportive care, 200 mL of CP was transfused twice at 2-day intervals. In addition, to break the severe macrophage activation syndrome-like immune response, tocilizumab was administered with the 2nd infusion. In the following period, the patient rapidly improved in terms of clinical and laboratory values. Timely infusion of CP in this patient, who had difficult comorbidities and immune compromisation and an uncontrolled immune response similar to macrophage activation syndrome, is a demonstrative example [26].

## 5. Conclusion 

In many countries, large-scale CP studies have started to be carried out, some of which have involved more than 1500 patients6U.S. National Library of Medicine (2020). ClinicalTrials.gov Database [online]. Website https://clinicaltrials.gov [accessed 00 Month Year].. In those studies comparing various comparators with immune plasma, many parameters, especially effectiveness and safety, were evaluated in terms of a wide range of clinical outcomes. Studies involving more than 100 patients are summarized in the Table. The results of those studies show the use of immune plasma and with which indications and timing it will be most beneficial. However, some of the most important publications on immune plasma effectiveness [27] and safety [28] are already at the preprint stage. The safety data in one study, which involved 5000 patients, indicated that immune plasma administration could be considered safe in those with severe or life-threatening diseases. In another study, in which both efficacy and safety were addressed, immune plasma treatment was evaluated as safe, and clinical improvement was achieved in 19 of 25 patients [27]. No significant adverse effect was observed in a study in which 26 patients were included in a center where the CP treatment was first applied in a country outside of China. Six of 17 patients who needed mechanical ventilation died, while none of the 9 patients who did not require mechanical ventilation died [29]. As more data from more studies accumulate, more robust conclusions will be drawn regarding immune plasma efficacy and safety.

**Table:** Table. Ongoing studies on immune plasma (CP: convalescent plasma) are listed by their key features. Among the studies registered on the ClinicalTrials.gov database, studies involving 100 or more patients were included. (CP: convalescent plasma, FMTVDM: Fleming method for tissue and vascular differentiation and metabolism; HCQ: hydroxychloroquine; ICU: intensive care unit; P: plasma; RL: Ringer’s lactate; SC: supportive care; WHO: World Health Organization).

Title	Country	Phase	Design	Major outcome measures	Number of cases	Estimated completion date
Efficacy and safety of novel treatment options for adults with COVID-19 pneumonia	Denmark	3	CP vs. Sarilumab vs. injective placebo, HCQ vs. Baricitinib vs. oral placebo (all with SC)	All-cause mortality or need of invasive mechanical ventilation	1500	June 2021
Convalescent plasma to limit SARS-CoV-2 associated complications	USA	2	CP vs. random P	Cumulative incidence of hospitalization or death prior to hospitalization, treatment-related serious adverse events and treatment-related grade 3 or higher adverse events	1344	January 2023
Convalescent plasma for hospitalized adults with COVID-19 respiratory illness (CONCOR-1)	Canada	3	CP vs. SC	Intubation or death in hospital	1200	December 2020
Passive immunity trial of nashville II for COVID-19	USA	3	CP vs. placebo	COVID ordinal outcomes acale by day 15	500	April 2021
Convalescent plasma vs. standard plasma for COVID-19	USA	1–2	CP vs. random P	28 day ventilator free days	500	August 2021
The Fleming [FMTVDM] directed CoVid-19 treatment protocol	USA	2–3	CP and 10 more treatment options	Improvement in FMTVDM measurement with nuclear imaging	500	November 2020
Convalescent plasma as therapy for covid-19 severe SARS-CoV-2 disease (CONCOVID study)	Netherlands	2–3	CP vs. SC	Overall mortality until discharge from the hospital or a maximum of 60 days after admission whichever comes first	426	July 2020
Convalescent plasma to limit COVID-19 complications in hospitalized patients	USA	2	CP vs. RL of saline	Percentage of subjects reporting each severity rating on WHO ordinal scale for clinical improvement	300	April 2023
convalescent plasma therapy vs. SOC for the treatment of COVID19 in hospitalized patients	Spain	2	CP vs. SC	Category changes in ordinal scale for hospitalization, mechanical ventilation etc.	278	July 2020
Convalescent plasma vs. placebo in emergency room patients With COVID-19	USA	2	CP vs. random P	Time to disease progression	206	December 2022
Evaluation of SARS-CoV-2 (COVID-19) antibody-containing plasma therapy	USA	3	CP vs. random P	Modified WHO ordinal scale (MOS) score: day 14	220	December 2021
Convalescent plasma collection and treatment in pediatrics and adults	USA	3	SC vs. 1 U CP vs. 2 U CP (according to disease severity)	Survival, morbidity reduction, length of stay in hospital	240	March 2021
Efficacy of convalescentplasma to treat COVID-19 patients, a nested trial in the CORIMUNO-19 cohort	France	2	CP vs. SC	Survival without needs of ventilator utilization or use of immunomodulatory drugs,WHO progression scale ≥6	120	June 2020
A phase II, open label, randomized controlled trial to assess the safety and efficacy of convalescent plasma to limit COVID-19 associated complications	India	2	CP vs. SC	Composite measure of the avoidance of - 1. Progression to severe ARDS (P/F ratio 100) and 2. All-cause mortality at 28 days	100	May 2021
Early transfusion of convalescent plasma in elderly COVID-19 patients. to prevent disease progression.	Italy	2–3	CP vs. SC	Rate of COVID-19 progression	182	June 2021
A study evaluating the efficacy and safety of high-Ttiter anti-SARS-CoV-2 Plasma in hospitalized patients with COVID-19 infection	USA	2	CP in ICU vs. CP in non-ICU	Overall mortality within 60 days, length of ICU stay	131	May 2023
Efficacy of human coronavirusimmune convalescent plasma for the treatment of COVID-19 disease in hospitalized children	Canada	2	CP vs. SC	Clinical recovery in 30 days	100	May 2022
Plasma therapy of COVID-19 in Critically ill patients	USA	2	CP vs. random P	Time to improvement	105	April 2021
Efficacy and safety human coronavirus immune plasma (HCIP) vs. control (SARS-CoV-2 nonimmune plasma) among adults exposed to COVID-19	USA	2	CP vs. random P	Cumulative incidence of composite outcome of disease severity	150	January 2023

In the near future, antibody-based treatments will not only constitute CP7U.S. Food and Drug Administration (2020). Coronavirus (COVID-19) Update: FDA Coordinates National Effort to Develop Blood-Related Therapies for COVID-19 [online]. Website https://www.fda.gov/news-events/press-announcements/coronavirus-covid-19-update-fda-coordinates-nationaleffort- develop-blood-related-therapies-covid-19 [accessed 20 June 2020].. Plans are in motion to purify polyclonal antibodies such as hyperimmune globulin (H-Ig) from pooled plasma of donors to develop an unbranded standard antibody product. With the production of hyperimmune globulins, an antibody-based treatment will not have to be stored locally but can be shipped to many locations worldwide. These types of treatment will also offer a standardized and highly efficient, scalable option, and clinical outcomes can be tracked more objectively. If the expected success is achieved in the previous experiences, then steps towards the production of recombinant antibodies in mammalian cell lines can be envisaged.
